# The Differentiation of Acute Broncho-Pneumonia and Bronchitis in Children

**Published:** 1893-12

**Authors:** Henry E. Tuley

**Affiliations:** Louisville, Ky.


### THE DIFFERENTIATION OF ACUTE BRONCHO-PNEUMONIA AND BRONCHITIS IN CHILDREN.

BY HENRY E. TULEY, M. D., LOUISVILLE, KY.

In the writings of most the old diagnosticians and in many of the more recent ones, bronchitis and acute broncho-pneumonia have been confounded, but it is the belief of the writer that many cases of bronchitis so diagnosticated are in truth cases of acute broncho-pneumonia.

My attention to this point was first aroused while resident at the New York Infant Asylum, during the service of Dr. L. Emmett Holt, and later by a comprehensive and thorough paper on this subject by Dr. Holt, published in the Archives of Pediatrics, in December, 1891.

The object of this short paper—for the facts of which I am indebted to above mentioned article, is to second Dr. Holt in his efforts, hoping to make plain the identity of many cases of bronchitis and acute broncho-pneumonia, especially that large class of cases termed "CAPILLARY BRONCHITIS."

A glance at our city death returns will show how prevalent among our medical brethren is the use of this term "capillary bronchitis."

It is evident without demonstration that it is impossible for an inflammation to occur in the smaller bronchi and their terminal branches, in an infant, without spreading to and involving the surrounding structures.

It is true that there are cases where it may be impossible clinically to make a differentiation but I believe the number of such is small.

There is little or no difficulty in making out a bronchitis of the larger bronchi—both from the physical signs, the character of the rales, etc., and the general symptoms, absence of great prostration, little fever, etc. It is rare that we find a uni-lateral bronchitis. The temperature in bronchitis is usually high during the first day, reaching its maximum on the first day, 102° or 103° which lasts twenty-four or thirty-six hours; a fall to 100° or normal then occurs, flluctuating for a degree or so for several days with a subsidence coincident with the cessation of the local trouble. There is little or no prostration and the symptoms of a severe lesion are absent.

But how different the clinical aspect of a broncho-pneumonia. Given a case with an initial temperature of 101° or thereabouts, and the subsequent history shows a rise on successive days to 102° or 103° with the general symptoms; prostration, cyanosis, etc., present, we are sure of having a serious disease of the lung to deal with, though the physical signs may not present themselves until later.

As to the importance of a diagnosis in these cases : I quote from Dr. Holt's article : "It may be urged that as there is no difference in the management of a case of severe bronchitis and one of broncho-pneumonia, a diagnosis is of no practical importance. This may be admitted from the standpoint of the therapeutist in the acute attack. It is, however, essential to a correct understanding of what so often follows such an attack ; relapses, recurrences, or chronic pneumonia, to know from the beginning there existed not only a bronchitis but broncho-pneumonia,"

Our diagnosis then is to be largely made up from the " Severity of all the general symptoms, cough, prostration, restlessness, dyspnoea, and cyanosis." If the physical signs of pneumonia be present, bronchial breathing, dullness, etc., there is no doubt about the diagnosis, but I have often seen cases in which at the autopsy, disseminated patches of broncho-pneumonia were found, but in which there had not been present dulness or bronchial breathing at careful physical examinations made before death. The temperature in broncho-pneumonia is variable, no typical course being followed. In the majority of cases it is high and remittent, in others it is 103° or below, but it is not infrequent that we have temperatures running below 101°, sometimes even sub normal ; the latter occurring more frevuently in children who are very delicate, than among those of robust condition. There is generally a morning remission and an evening exacerbation but it is not unusual to to meet with the opposite condition, a higher morning temperature lasting for several days, the resulting chart being very characteristic. The temperature may reach as high as 107° or 108° and is generally fatal. The highest temperature recorded in Dr. Holt's series of cases with recovery was 106°, of twenty cases, 75 per cent. proving fatal.

The duration of the temperature is of course very varied, cases are reported as lasting from a few days to fifty-one days or more; the greatest mortality in the above referred to cases being 100 per cent. in those cases lasting four days or less.

Of the physical signs which we may expect in pneumonia are dulness, bronchial breathing or change in the respiratory note, rales and pleuritic friction sounds. The absence of dulness and bronchial breathing, as before stated, does not by any means preclude the presence of pneumonia--the patches of consolidation may be so disseminated with normal pulmonary tissue intermixed as to cause no deviation from the norma pulmonary resonance, or change in the respiratory note, except perhaps a slight weakening of the latter. Time and again have I seen cases throughout have no dulness on superficial or deep percussion, with the other pathognomonic signs of pneumonia present.

The presence and character of the rales and particularly their location is of importance. Finding a localized area of rales either on one side or both sides should cause pneumonia to be suspected, especially if over one or both lower lobes behind.

Of pleuritic sounds Dr. Holt says—"They may be looked for in broncho-pneumonia whenever we have large areas of consolidation present, but in my experience, based upon post mortem examination, and it has not been small, almost never under other circumstances. In the cases under discussion they are so rarely heard that they may then be dropped from our thoughts altogether."

The general aspect of the child when afflicted with pneumonia is characteristic, the prostration is generally severe, the cyanosis more or less marked, dilation of the alae nasi present increase in the pulse and respiration in the proper ratio, and we nearly always hear the "pneumonia sign" caused by the short, sharp respiration. It is the combination of these general symptoms which in the absence of pathognomonic signs would cause us to class these cases as broncho-pneumonia, and not "capillary bronchitis," especially those cases which only give signs of generally distributed rales with few other definite signs. The diagnosis can well be verified by an autopsy if it may come to that.

Broncho-pneumonia may always be considered a dangerous disease, the percentage of mortality has been variously stated. Holt gives it at 68 per cent. in his 156 cases recorded, while the mortality from acute bronchitis is practically *nil*.

In conclusion I would state that in the opinion of the writer, the term capillary bronchitis cannot properly be used, for pathologically, and in the great majority of cases clinically, these are cases of acute broncho-pneumonia, and not "capillary bronchitis."

